# Dynamic Changes in the Gut Microbiota During Peripartum in Jennies

**DOI:** 10.3390/ani15091337

**Published:** 2025-05-06

**Authors:** Xinyue Wang, Yang Shao, Xiaoling Zhou, Zheng Li, Jingze Liu, Mingyao Tang, Yixin Yang, Liang Deng

**Affiliations:** 1 Department of Animal Genetics, Breeding and Reproduction, College of Animal Science and Veterinary Medicine, Shenyang Agricultural University, Shenyang 110866, China; 2022240704@stu.syau.edu.cn (X.W.); 2022220555@stu.syau.edu.cn (Y.S.); 2019220503@stu.syau.edu.cn (Z.L.); 2020240537@stu.syau.edu.cn (J.L.); 2021240611@stu.syau.edu.cn (M.T.); 2021240628@stu.syau.edu.cn (Y.Y.); 2 Department of Pratacultural Science, College of Animal Science and Technology, Tarim University, Alaer 843300, China; zxldky@126.com

**Keywords:** donkey, *Equus asinus*, female donkeys, gut microbial colonization, peripartum period, 16S rRNA

## Abstract

The peripartum period is critical for breeding female donkeys (i.e., jennies) and ensuring the delivery of healthy neonatal foals. The gut microbiota deeply influences the host metabolism. This study aimed to investigate the dynamic changes in the gut microbiome during the peripartum period in jennies. Fresh fecal samples of eight adult jennies were collected at the following seven sampling time points: 21, 7, and 3 days prepartum (G21, G7, and G3) and 1, 3, 7, and 14 days postpartum (L1, L3, L7, and L14). Sequencing of the V4 hypervariable regions of the 16S rRNA genes was carried out using fecal samples to identify the differences in the microbiome across the peripartum period. Bacteroidota and Firmicutes were the most abundant bacterial phyla in the feces. *Treponema* and *Lachnospiraceae XPB1014 group* significantly increased in the L3 group compared to the G7 group (*q* < 0.05), and a decline trend was observed in L1 group around parturition. The genus *Clostridium sensu stricto 1*, family Clostridiaceae, and order Clostridiales were considered to be biomarkers of the L3 group. Among the 25 functional pathways detected by Kyoto Encyclopedia of Genes and Genomes pathway analysis, beta lactam resistance, insulin resistance, and peptidases were the top three important pathways observed in the gut microbiota during the peripartum period in jennies. The gut microbial structure changed significantly at different time points during the peripartum period in jennies. These results contribute to a better understanding of the gut microbiota to ensure health care during important phases from late pregnancy to early lactation in jennies.

## Simple Summary

Profound alterations occur in maternal physiology throughout the late pregnancy, parturition, and the neonatal periods in jennies (i.e., female donkeys). These alterations may have direct effects on the early microbial colonization of the foal’s gut. In this study, seven time points, both prepartum and postpartum, were used to investigate the changes in the gut microbiome of jennies using the next-generation sequencing of 16S rRNA. The key findings show that the gut microbial structure changed significantly at different time points during the peripartum period in jennies. Understanding these microbial dynamics can enhance the ability to support the health of jennies and their foals.

## 1. Introduction

The gut microbiome is an “additional genome” of the host and is associated with a host’s health via multiple pathways [1]. The gut microbiota exhibits remarkable diversity and unique functional characteristics, playing an important role in host development, metabolism, and immunity [2]. To date, microbiomic analysis has been widely used to better define the intestinal microbiota and its metabolic and immunoregulatory contributions to health and disease [3].

The hindgut of equines includes the cecum, colon, and rectum, which are rich in bacteria, fungi, and other microorganisms, providing the host with important raw materials, including microbial-derived proteins and fermentable sugars (e.g., glucose and xylose) released from plant polysaccharides [4]. The species and quantity of the gut microbiota of donkeys are affected by diverse factors, including *Equus* species, digestive tract segments, geographical regions, environmental conditions, and diet composition. In different *Equus* species, such as Shetland Pony, Mongolian Wild Ass, and Plain Zebra, the intestinal bacterial community composition and diversity differed significantly; however, their metabolic gene functions were similar [5]. The domesticated donkey (*Equus asinus*) fecal microbiota differed from that of pony and their derived hybrids, which was related to a higher relative abundance and diversity of taxa with known roles in plant material degradation [6]. The complete microbiome spectrum and annotated functions of several digestive tract segments of donkeys have been revealed [3]. The donkey microflora’s structure and function showed significant differences in different geographical regions [1]. The gut microbiota of Tibetan wild ass (*Equus kiang*) appeared to have undergone changes to adapt to changes in the external environment [7]. Dietary supplementation with yeast polysaccharide stimulated the growth-promoting gut microbiota (*Lactobacillus* and *Prevotella*), and had no adverse effects on the metabolism of both Dezhou donkey jennies and foals [8].

The peripartum period is critical for breeding jennies and ensuring the delivery of healthy neonatal foals. Profound alterations occur in the maternal physiology through late pregnancy, parturition, and the neonatal period [9]. These alterations may have direct effects on the early microbial colonization of the foal’s gut, which is crucial for the health of the neonatal foals [10]. An appropriate diet for jennies during late pregnancy increased the antioxidant capacity and reduced inflammatory cytokine levels, which were associated with alterations in the gut microbiota’s composition [11]. The nutrient requirements of jennies greatly increases in the last trimester of pregnancy, and improved understanding of the metabolic profile during the peripartum period might assist in monitoring the health status of jennies [12]. Changes in the fecal bacterial and plasma metabolism of donkeys throughout pregnancy have been reported, which indicate that host–bacteria interactions during the gestation period influence host metabolism [13].

To the best of our knowledge, little is known about the changes to the gut microbiota throughout the integrated peripartum period in jennies. Therefore, we hypothesized that the gut microbiome would change dramatically pre- and postparturition, owing to the increased metabolic demands and oxidative stress of jennies. Herein, seven time points, from the prepartum to postpartum periods, were used to investigate the changes in the gut microbiome in jennies using next-generation sequencing of 16S rRNA. The results will improve understanding of the gut microbiota in maintaining a mutual symbiotic relationship with the host in the peripartum period.

## 2. Materials and Methods

### 2.1. Animal Welfare Statement

Animals were obtained, kept, and used with the approval of the Animal Care and Use Committee of Shenyang Agricultural University (approval no. 202103021).

### 2.2. Animals

The research was performed between April and June of 2021. All jennies originated from Liaoxi donkeys and were kept under identical feeding conditions at a breeding facility located in Liaoning Province, China. The population of donkeys comprised eight female donkeys, each with an average live weight of 283.6 ± 43.2 kg, age of 5.4 ± 1.3 years, and parities of 1.8 ± 0.4, delivered within four weeks. These animals were continuously tracked to monitor the dynamic development of the gut microbiota from 21 days before foaling to 14 days after foaling. The diet comprised 65% dry corn stover and 35% commercial concentrate supplemented with major and trace elements (commercial Mutianli donkey feed, Xinjiyuan Ltd. Co., Shenyang, China). A nutritional analysis was conducted on samples of dry corn stover and the commercial concentrate (Table 1). The dry corn stover was provided to the jennies four times each day (5:30, 12:30, 18:30, and 23:30), and a total of 2.5 kg of the commercial concentrate was administered to each jenny three times throughout the day (5:30, 12:30, and 18:30). Daily feed amounts were adjusted to satisfy or surpass the nutritional needs of the 300 kg jennies (referred to pony mares) in alignment with the guidelines established by the National Research Council: Donkeys and other equids [14]. The animals had constant access to fresh water. The jennies were kept in good health, as evaluated following the recommended standards of The Donkey Sanctuary [15]. The body condition score (1 = poor, 2 = moderate, 3 = ideal, 4 = fat, and 5 = obese) of jennies was 2.7 ± 0.5 during this study. After delivery, the foals underwent assessments for their health and viability based on established guidelines [16].

### 2.3. Sampling

For all jennies, fresh fecal samples were collected at seven sampling time points: 21, 7, and 3 days prepartum (G21, G7, and G3) and 1, 3, 7, and 14 days postpartum (L1, L3, L7, and L14). All fecal samples were collected between 9:00 and 10:00 a.m. Fecal material from each jenny was obtained by rectal palpation. Approximately 5 g of feces were collected from the interior of fecal balls, avoiding contamination. The samples were stored in sterilized tubes and kept at a refrigerator of −80 °C until DNA extraction.

### 2.4. Samples’ Processing and 16S rRNA High-Throughput Sequencing

DNA was extracted from the total genomic material of each fecal sample utilizing the method of cetyltrimethylammonium bromide [17]. The concentration and purity of the DNA were assessed with 1% agarose gel electrophoresis post-extraction. Subsequently, the DNA was adjusted to a concentration of 1 ng/µL with sterile water. Then, high-throughput sequencing of the 16S rRNA was conducted to analyze the diversity and composition of the bacterial community.

The amplification of the V4 hypervariable region of 16S rRNA genes was achieved with the use of a primer pair consisting of 515F (5’-GTGCCAGCMGCCGCGGTAA-3’) and 806R (5’ GGACTACHVGGGTWTCTAAT-3’) [18]. A positive template was used alongside a negative control, which consisted of distilled water. Subsequent to the PCR amplification, the resulting products underwent electrophoresis and were subsequently purified using an AxyPrep DNA Gel Extraction Kit (Axygen Biosciences, Union City, CA, USA) following the guidelines provided by the manufacturer. The PCR products were assessed in terms of concentration, utilizing a Quantus Fluorometer (Promega, Madison, WI, USA). The purified amplicons were pooled, followed by sequencing in both directions on the Illumina MiSeq PE300 and NovaSeq PE250 platforms (Illumina, San Diego, CA, USA). The raw data for the 16S gene sequences underwent quality filtration implemented by Fastp (v 0.20.0, https://github.com/OpenGene/fastp) (accessed on 5 September 2022). Merging of the sequencing reads was performed using FLASH (v 1.2.7, http://ccb.jhu.edu/software/FLASH/) (accessed on 5 September 2022). The clustering of operational taxonomic units (OTUs) was performed at a 97% similarity level using UPARSE (v 7.0.1001, http://drive5.com/uparse/) (accessed on 21 September 2022). The sequences representing each OTU taxon were analyzed using QIIME (v 1.9.1, http://qiime.org/scripts/split_libraries_fastq.html) (accessed on 25 September 2022), and the representative sequence were evaluated using the Silva rRNA database (v 138, http://www.arb-silva.de/) (accessed on 26 September 2022) with the Ribosomal Database Projection Classifier (v 2.2.0) [19].

### 2.5. Bioinformatics and Statistical Analysis

An analysis of the alpha diversity was carried out, which included metrics such as the Chao1 and ACE indices to reflect the richness (i.e., number of taxonomic groups) alongside the Shannon and Simpson indices, providing insight into the diversity (i.e., the variety and quantity of taxonomic groups). This assessment was executed utilizing QIIME (v 1.9.1). In QIIME, the assessment of the community structure was performed by calculating the beta diversity index, utilizing UniFrac and Bray–Curtis distances to evaluate the differences in bacterial community compositions across various samples. Non-metric multidimensional scaling (NMDS) and analysis of similarity (ANOSIM) were performed for all samples, utilizing the count-based Bray–Curtis distance calculated at the OTU level. The analysis was carried out utilizing SPSS v 22.0 for data processing (IBM Corp., Armonk, NY, USA). The analysis of variations in microbial community abundance among the different groups was conducted utilizing Metastat statistical methods. The evaluation of the significant differences in abundance among the various groups was conducted by multiple hypothesis tests for sparsely sampled features and correction for false discovery rates. Linear discriminant analysis (LDA) coupled with effect size (LEfSe) was carried out to identify the most differentially abundant taxa among the different groups (LDA > 3). The abundances of the functional categories within the Kyoto Encyclopedia of Genes and Genomes (KEGG) ortholog database were estimated through the application of the Phylogenetic Investigation of Communities by Reconstruction of Unobserved States (PICRUSt) [20]. A random forest analysis was carried out utilizing the relative abundance of gut microbiota present in each sample using varSelRF (v 0.7.8, https://cran.r-project.org/web/packages/varSelRF/index.html) (accessed on 29 September 2022). Statistical significance was set at *p* < 0.05 and *q* < 0.05.

## 3. Results

### 3.1. Sequencing Information

**Figure 1:**
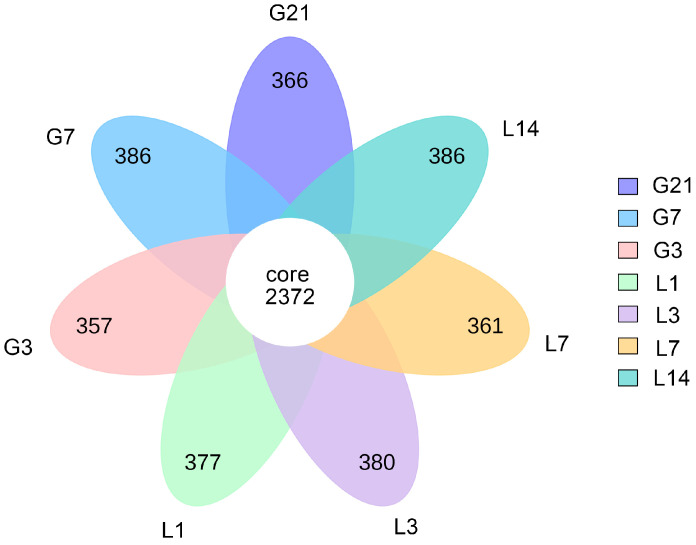
The flower diagram based on the OTUs’ distribution between fecal samples G21, G7, G3, L1, L3, L7, and L14 from jennies. Different colors represent different groups. G21, 21 days prepartum; G7, 7 days prepartum; G3, 3 days prepartum; L1, 1 day postpartum; L3, 3 days postpartum; L7, 7 days postpartum; and L14, 14 days postpartum.

Approximately 4,507,425 reads were analyzed from the 56 fecal samples (i.e., bacterial communities). The total number of reads, average length of the reads, and number of base pairs obtained from the original file of each fecal sample before and after quality control filters are presented in: Table S1. Approximately 7093 OTUs were identified by clustering at a 97% sequence similarity. Representative sequences from those OTUs were assigned to 41 bacterial phyla (Table S2). The number of OTUs shared by each group of samples was 2372. There were 366, 386, 361, 380, 377, 357, and 386 unique OTUs identified in the G21, G7, G3, L1, L3, L7, and L14 groups, respectively (Figure 1).

### 3.2. Taxonomic Composition of the Bacterial Communities

**Figure 2:**
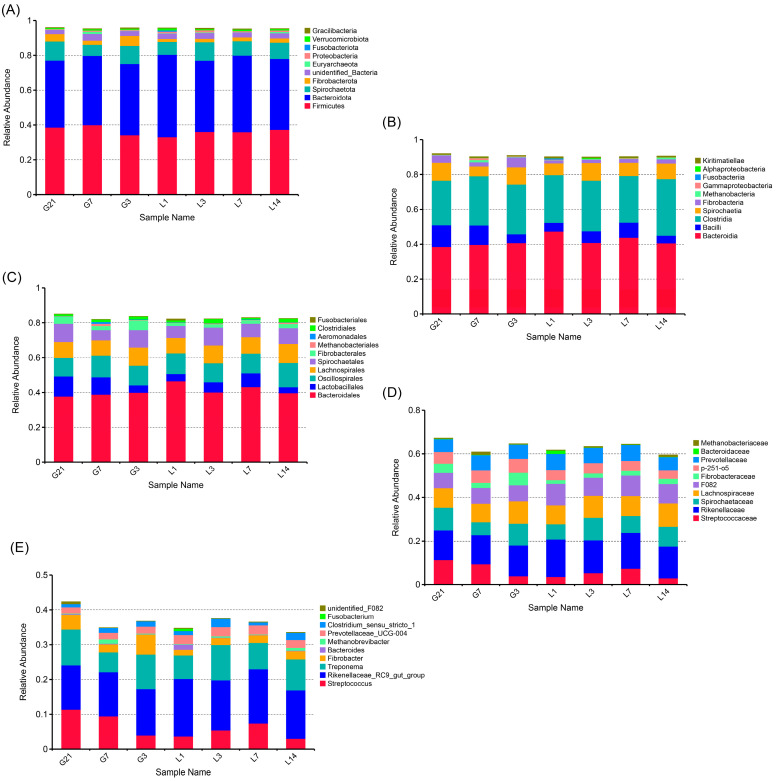
Polymerase chain reaction amplification and 16S rRNA pyrophosphate sequencing were utilized to analyze the fecal samples from the G21, G7, G3, L1, L3, L7, and L14 jennies, revealing the bacterial composition: (**A**) composition of bacteria at the phyla level of the fecal samples; (**B**) composition of bacteria at the class level of the fecal samples; (**C**) bacterial composition at the order level of the fecal samples; (**D**) bacterial composition at the family level of the fecal samples; (**E**) composition of the bacteria at the genus level of the fecal samples. G21, 21 days prepartum; G7, 7 days prepartum; G3, 3 days prepartum; L1, 1 day postpartum; L3, 3 days postpartum; L7, 7 days postpartum; and L14, 14 days postpartum.

Fecal microbial communities during the peripartum period in jennies at the level of bacterial phyla, class, order, family, and genus were analyzed. Bacteroidota and Firmicutes were the most abundant bacterial phyla in the feces (Figure 2A). The top 10 species at the class, order, and family levels are shown in Figure 2B–D. At the genus level, the top 10 abundant genera were *Rikenellaceae RC9 gut group*, *Treponema*, *Streptococcus*, *Fibrobacter*, *Bacteroides*, *Methanobrevibacter*, *Prevotellaceae UCG-004*, *Clostridium sensu stricto 1*, *Fusobacterium*, and *unidentified F082* (Figure 2E).

### 3.3. Alpha Diversity

**Figure 3:**
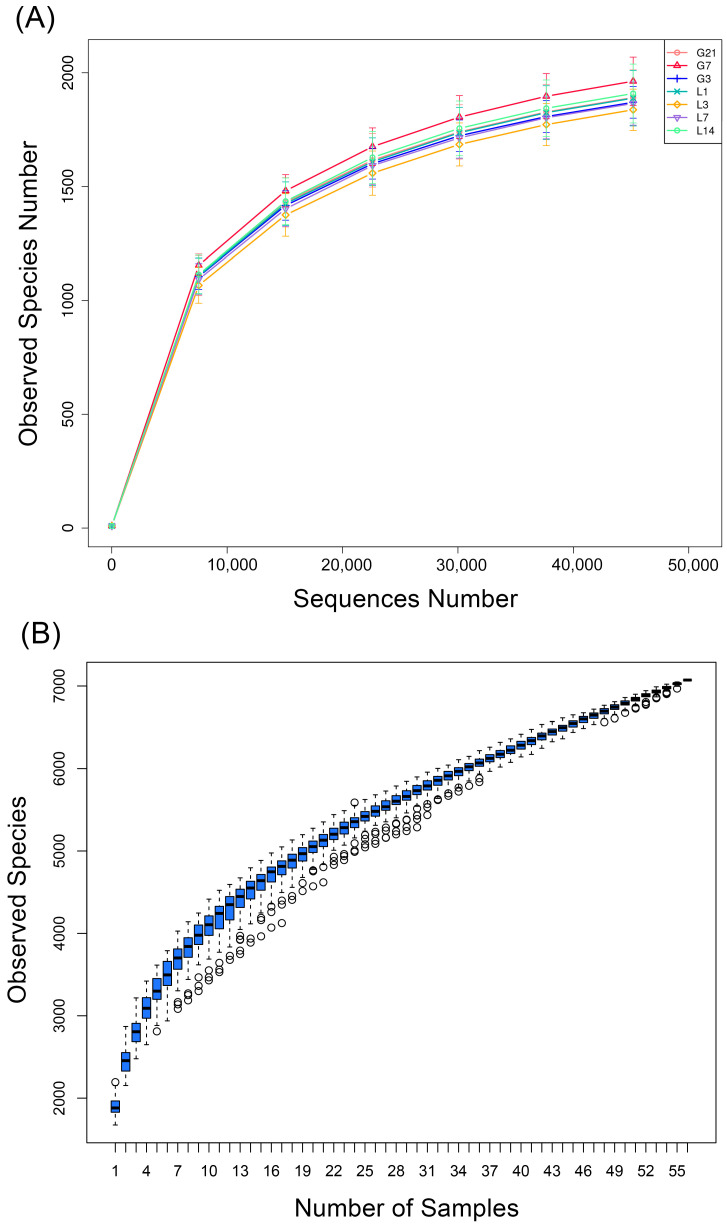
Alpha diversity indices in fecal samples G21, G7, G3, L1, L3, L7, and L14 from jennies during the peripartum period: (**A**) rarefaction curves; (**B**) species accumulation curve. Open circles represent the outliers. G21, 21 days prepartum; G7, 7 days prepartum; G3, 3 days prepartum; L1, 1 day postpartum; L3, 3 days postpartum; L7, 7 days postpartum; and L14, 14 days postpartum.

Figure 3A illustrates a gradual flattening of the rarefaction curve, which suggests that the sequencing data is adequate for the analytical purposes of this research. The samples’ rarefaction curves began to level off around 10,000 sequences, suggesting that the sequencing depth was adequate to capture a majority of the species present in the samples. The box plot in Figure 3B exhibited a tendency to level off with the increase in sample size, further confirming the adequacy of the sample size. Community richness of the samples was assessed through the OTUs found in each individual sample, as depicted in Table S3.

### 3.4. Beta Diversity

**Figure 4:**
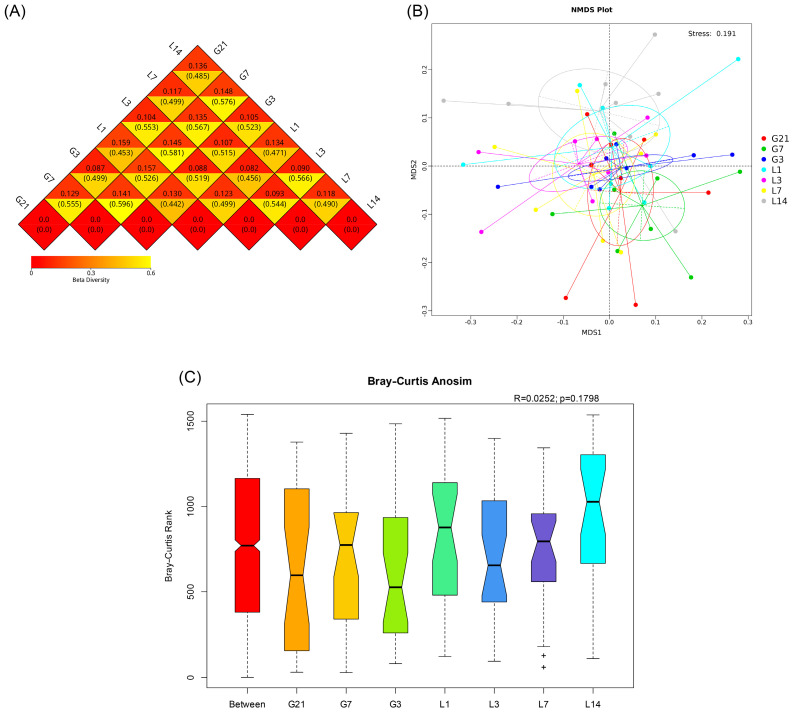
Investigation into the variations in the beta diversity of jennies during the peripartum period. (**A**) Heatmap depicting both weighted and unweighted UniFrac distances. Each square contains a numerical value that signifies the difference coefficient between the two samples. Within each square, the values above and below denote the weighted and unweighted UniFrac distances, respectively. (**B**) A plot demonstrating non-metric multidimensional scaling. (**C**) Evaluation of the similarity among the bacterial communities in the samples utilizing Bray–Curtis dissimilarity. Black pluses represent the outliers. G21, 21 days prepartum; G7, 7 days prepartum; G3, 3 days prepartum; L1, 1 day postpartum; L3, 3 days postpartum; L7, 7 days postpartum; and L14, 14 days postpartum.

To compare the differences in bacterial community composition during the peripartum period in jennies, weighted and unweighted UniFrac distances were calculated (Figure 4A). The results show that differences existed during the peripartum period among the seven groups (G21, G7, G3, L1, L3, L7, and L14). The differences in gut microbiota from the jennies across the peripartum period were identified using NMDS. The obtained results suggest that the microbial species separated during the peripartum period (Figure 4B). In addition, ANOSIM based on the Bray–Curtis distance (R = 0.0252, *p* = 0.1798) shows that the inter-group differences were slightly but not significantly greater than the intra-group differences (Figure 4C).

### 3.5. MetaStat Analysis

**Figure 5:**
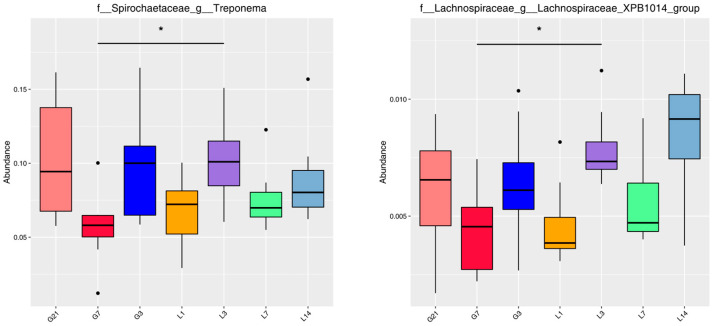
MetaStat analysis at the genus level for gut microbiota during the peripartum period in jennies. * Indicates a significant difference (*q* < 0.05). Black circular dots represent the outliers. G21, 21 days prepartum; G7, 7 days prepartum; G3, 3 days prepartum; L1, 1 day postpartum; L3, 3 days postpartum; L7, 7 days postpartum; and L14, 14 days postpartum.

MetaStat analysis was used to identify the taxa with significant inter-group variation during the peripartum period in jennies. At the genus level, the *q*-value was less than 0.05 only for the *Treponema* and *Lachnospiraceae XPB1014 group* among the multiple groups. Furthermore, the abundance of these two genera significantly increased in the L3 group compared to the G7 group (*q* < 0.05), and a decline trend was observed in the L1 group around parturition (Figure 5).

### 3.6. LDA Effect Size (LEfSe) Analysis

**Figure 6:**
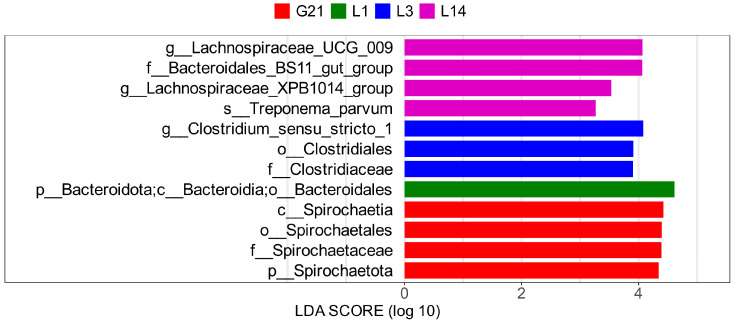
Taxonomic biomarkers found in the gut microbiota between different groups during the peripartum period in jennies, according to the linear discriminant analysis (LDA) effect size analysis. Statistically significant groups were reported with LDA scores > 3. G21, 21 days prepartum; L1, 1 day postpartum; L3, 3 days postpartum; and L14, 14 days postpartum.

To further identify the microbial biomarkers of the gut microbiota among the seven groups of jennies during the peripartum period, an LEfSe analysis was applied (Figure 6). Notably, several taxa with evolutionary relationships were considered to be biomarkers of the L3 group, such as the genus *Clostridium sensu stricto 1*, family Clostridiaceae, and order Clostridiales.

### 3.7. Functional Prediction

**Figure 7:**
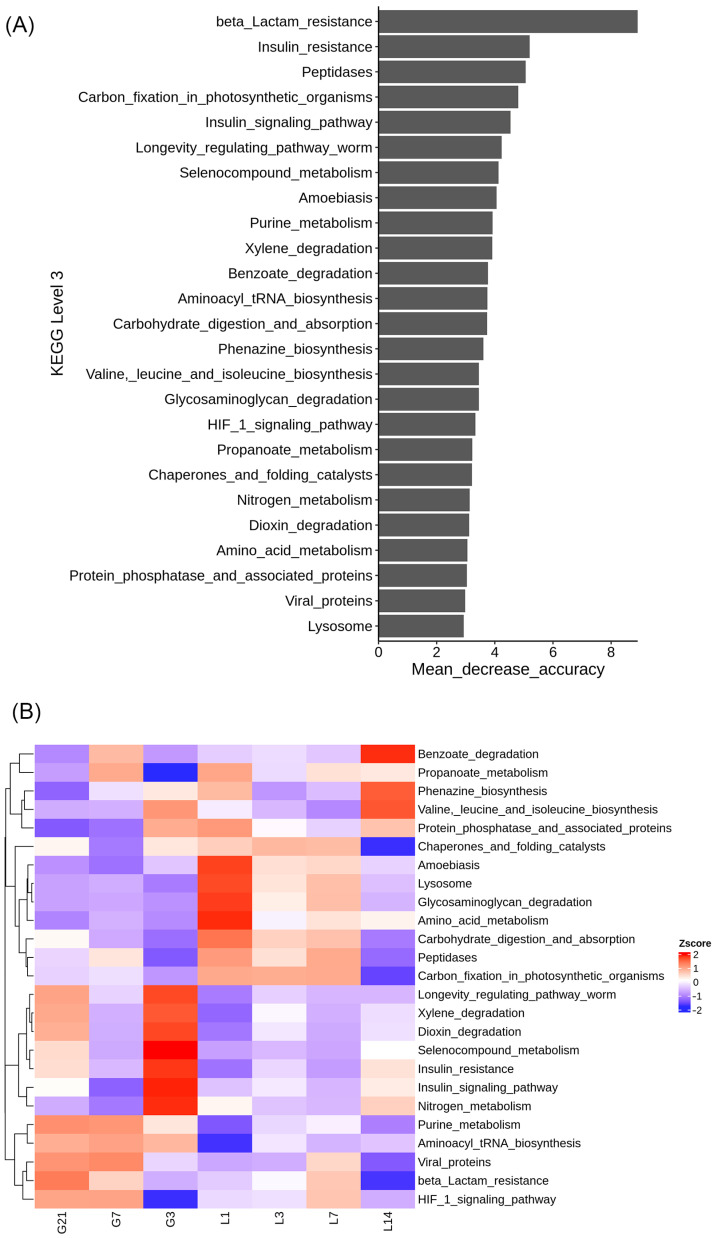
Prediction of bacterial functions throughout the perinatal period: (**A**) functions of the top 25 bacteria identified throughout the peripartum period in jennies; (**B**) distribution of the functions of top 25 bacteria throughout the peripartum period in jennies. G21, 21 days prepartum; G7, 7 days prepartum; G3, 3 days prepartum; L1, 1 day postpartum; L3, 3 days postpartum; L7, 7 days postpartum; and L14, 14 days postpartum.

The predicted functions affected by the bacteria represented by the 16S rRNA data, as analyzed using PICRUSt, were ranked according to importance from the highest to the lowest. Among the 25 functional pathways detected at the KEGG pathway level 3, beta lactam resistance, insulin resistance, and peptidases were the top three important pathways predicted to be affected by the gut microbiota during the peripartum period in jennies (Figure 7A). During the prepartum period, the longevity regulating pathway worm, xylene degradation, dioxin degradation, selenocompound metabolism, insulin resistance, insulin signaling pathway, and nitrogen metabolism pathway were enriched compared with those in the postpartum period. By contrast, amino acid metabolism; glycasaminoglycan degradation; lysosome; amoebiasis; valine, leucine and isoleucine biosynthesis; phenazine biosynthesis; and benzoate degradation pathways were enriched during the postpartum period (Figure 7B).

## 4. Discussion

Herein, 16S rRNA gene sequencing technology was used to identify the bacterial species constituting the gut microbiota of jennies at seven different time points during the perinatal period. The analyses determined that remarkable changes in the microbiota occur during the perinatal period in jennies. Understanding these changes is essential to comprehensively characterize gut microbiota in jennies from a breeding perspective. This study provides further evidence supporting the important influences of parturition and lactation on the microbial community’s structure and richness of jennies.

In the present study, 2372 OTUs were common among the seven groups. These OTUs could play an important role in the stability and function of intestinal microecological environment in jennies. Among these OTUs, some were only identified at 1 day postpartum, and are probably related to the occurrence of parturition. In mares, the hindgut microbiota was proven to be altered by parturition [21]. Accordingly, it was suggested that parturition might disrupt an optimal and permissive environment for the growth and reproduction of gut microbiota in jennies, leading to greater microbial variability and richness [22].

This study shows that Bacteroidota and Firmicutes were the most abundant bacterial phyla in the feces, and the average relative abundance of Bacteroidota was higher than that of Firmicutes in jennies during the perinatal period. Similar results were obtained in a previous study of the fecal microbiota of healthy horses [23]. Bacteroidota is the main microbial phylum for carbohydrate metabolism in herbivores and is important for improving disease prognosis [3]. The high abundance of Firmicutes might be related to the anatomical physiology and feeding habits of donkeys, which mainly ingest insoluble fiber and use the cecum and large colon as the main sites for fermentation [24]. The Bacteroidota/Firmicutes ratio observed in the results might also be due to the bacterial DNA extraction method used [25]. The levels of Bacteroidota and Firmicutes remained relatively stable during the perinatal period, which is in line with previous studies in donkeys [13,22].

The present results demonstrate that the relative abundances of *Streptococcus*, *Treponema*, and *Lachnospiraceae XPB1014 group* changed during the perinatal period at the genus level of the gut microbiota. Previous studies have demonstrated pregnancy-induced alterations in the gut bacterial composition [26]. Diverse bacteria provide many metabolic capacities and functional redundancy in pregnant donkeys, ensuring a sufficient supply of nutrients for maternal health and fetal development [13]. Herein, the relative abundance of *Streptococcus* gradually decreased from 21 days prepartum to 1 day postpartum. *Streptococcus*, previously observed in the horse gastrointestinal tract, is known to be a starch-utilizing genus and is also considered beneficial to health because of its complex interaction with the host [22]. The *Streptococcus* strains isolated from donkey intestines were able to grow on pyruvate and did not produce acid from sucrose and raffinose [27]. However, some reports indicate that *Streptococcus* is an important cause of infectious diseases [28,29]. It has been suggested that the gradual decrease in the abundance of *Streptococcus* in jennies until the first day postpartum might be caused by the decrease in crude fiber intake resulting from parturition stress.

According to the taxonomic composition at the genus level and the MetaStat analysis, the abundance of genus *Treponema* was relatively lowest on 1 day postpartum around parturition, which is similar to the trends observed during the prenatal period in sows [30]. *Treponema* spp. are spiral-shaped bacteria of the phylum Spirochaetes [31]. *Treponema* is highly abundant in the cecum and closely associated with cellulose digestion and utilization, and hemicellulose degradation. *Treponema* was also positively associated with lipid-metabolism-related genes [32]. Our previous study revealed that early lactating jennies showed significantly lower serum levels of triglycerides than late pregnant jennies [12]. Therefore, it was suggested that the decrease in *Treponema* levels on the first day postpartum was related to the dramatic increase in energy consumption for milk yield after foaling in jennies [33].

In this study, the MetaStat analysis reveals that the abundance of genus *Lachnospiraceae XPB1014 group* was relatively lower on the first day postpartum compared with that at 3 days postpartum in jennies. In a previous study, postpartum mares had a corresponding decrease in the relative abundance of the family Lachnospiraceae compared with that in prepartum mares [34]. These significant differences in Lachnospiraceae family levels have been confirmed among different equines such as donkey, ponies, and inter-species hybrids [6]. Lachnospiraceae have been associated with maintaining gut health and correlated strongly and negatively with intestinal inflammation [22]. Lachnospiraceae exhibited anti-inflammatory functions and were also reported to be involved in the production of short-chain fatty acids [35,36]. This suggests that Lachnospiraceae play a key role in maintaining gut health during the perinatal period in jennies.

At the genus level, the genus *Clostridium sensu stricto 1* might be a biomarker at 3 days postpartum. *Clostridium sensu stricto 1* was commonly considered to be an opportunistic pathogenic bacteria of the animal intestine [37]. With the increasing relative abundance of *Clostridium sensu stricto 1*, inflammatory factors were secreted [38]. The abundance of *Clostridium sensu stricto 1* was positively correlated with the levels of biomarkers of systemic low-grade inflammation in Dezhou Donkeys [22]. This result indicates that the jennies exhibit some inflammation during the early lactating period. The observed inflammation may serve as physiological evidence of tissue repair mechanisms, reflecting the body’s need to recover from placental-detachment-induced bleeding and adapt to reduced abdominal volume after parturition [39].

The functional KEGG pathways, such as the insulin resistance and insulin signaling pathways, were obviously highly enriched in the fecal microbiome during the prepartum period in the jennies. The prevalence of dyslipidemias is higher in donkeys (10–20%) than in horses and ponies, which is more evident in obese, pregnant, lactating, and older animals, perhaps because of the reduced insulin sensitivity [40]. Triglyceride metabolism has been suggested to be an important factor contributing to the pathogenesis of hyperlipidemia during the peripartum period in jennies [41]. Many members of the gut microbiota regulate lipid metabolism, and host immunity. The microbial communities can also harbor diverse antibiotic resistance genes, including those encoding β-lactam, tetracycline, aminoglycoside, and macrolide resistance [42,43]. Bacteroidota and Firmicutes mediate insulin resistance through modulation of glucagon-like peptide-1 secretion in obesity [44]. This suggests that the gut microbiota is deeply involved in insulin homeostasis and lipid metabolism in prepartum jennies.

The selenocompound metabolism pathway was significantly enriched in the fecal microbiome at 3 days prepartum in jennies. Our previous findings showed substantially lower serum selenium (Se) in lactating jennies than in pregnant jennies [12]. After foaling, the transport of selenocompounds is enhanced by the mammary epithelium from blood to milk for lactation.

## 5. Conclusions

The gut microbial structure changed dramatically within a few days pre- and postparturition, and it is essential for maintaining the gut health of jennies. These results contribute to a better understanding of the gut microbiota for ensuring health care during important phases, from late pregnancy to early lactation, in jennies. In particular, the specific relationship between the gut microbiota and metabolic profiles in the early lactation period of jennies requires further investigation. It could provide insight into how maternal gut microbiota influence the health and development of the neonatal foal.

## Figures and Tables

**Table 1:** Nutrient composition of the diet on a dry matter basis.

		Source	
Item	Corn Stover	Concentrate	Total
Dry matter (%)	88.82	88.27	88.64
Crude protein (%)	9.02	18.77	15.13
Crude fiber (%)	32.30	5.81	20.64
ADF (%)	39.33	11.82	25.68
NDF (%)	63.78	23.51	48.43
Ether extract (%)	1.43	4.13	2.78
Calcium (%)	0.31	1.13	0.95
Phosphorus (%)	0.10	0.81	0.52
Magnesium (%)	3.02	0.55	2.08
DE (MJ/kg)	7.53	12.23	9.22

ADF, acid detergent fiber; NDF, neutral detergent fiber; DE, digestible energy.

## Data Availability

The data presented in this study are available upon request from the corresponding authors.

## Supplementary Materials

The following supporting information can be downloaded at: https://www.mdpi.com/article/10.3390/ani15091337/s1, Table S1. Data preprocessing statistics and quality control information. Table S2. OTUs clustering statistics and representative sequences. Table S3. Number of OTUs per group and estimate of sequence diversity and richness.
