# Supplementary material for: Dynamic Changes in the Gut Microbiota During Peripartum in Jennies
Source: Animals (Basel). 2025 May 6;15(9):1337. doi: 10.3390/ani15091337 (PMC12071091; doi:10.3390/ani15091337)
Supplement: Supplementary file 1 [file animals-15-01337-s001.zip › animals-349475 supplementary Table S3 conversion.pdf]

animals

Table S3. Number of OTUs per group and estimate of sequence diversity and richness.

Sample	OTUs	Shannon	Simpson	Chao1	ACE	Goods Coverage
G21.1	2114	8.962	0.995	2280.081	2320.217	0.992
G21.2	1940	8.942	0.995	2131.73	2147.09	0.993
G21.3	1907	8.775	0.993	2081.299	2108.877	0.993
G21.4	1937	8.867	0.991	2127.785	2156.383	0.993
G21.5	1881	8.557	0.99	2059.955	2094.211	0.993
G21.6	1656	6.578	0.863	1835.474	1904.638	0.993
G21.7	1894	8.723	0.992	2075.96	2099.3	0.993
G21.8	1799	7.225	0.92	1969.205	2002.577	0.993
G7.1	1982	8.587	0.987	2169.6	2204.949	0.993
G7.2	1919	8.877	0.995	2083.903	2125.963	0.993
G7.3	1894	8.626	0.987	2070.609	2106.147	0.993
G7.4	2153	8.893	0.989	2356.208	2377.579	0.992
G7.5	1854	8.731	0.99	2041.041	2049.038	0.993
G7.6	1988	7.932	0.958	2172.116	2249.177	0.992
G7.7	1826	8.199	0.967	1993.476	2027.458	0.993
G7.8	2087	8.772	0.992	2298.589	2361.899	0.991
G3.1	1992	8.724	0.989	2153.762	2182.301	0.993
G3.2	1897	8.859	0.993	2073.734	2091.423	0.993
G3.3	1831	8.878	0.994	2001.83	2011.025	0.994
G3.4	1762	8.472	0.991	1932.205	1979.455	0.993
G3.5	1842	8.34	0.986	2021.465	2068.184	0.993
G3.6	1956	8.787	0.994	2149.574	2181.178	0.992
G3.7	1853	8.847	0.994	1996.011	2031.66	0.994
G3.8	1829	8.29	0.988	2080.333	2084.504	0.992
L1.1	1777	8.295	0.988	1907.052	1955.944	0.994
L1.2	2094	8.854	0.994	2304.384	2331.072	0.992
L1.3	1828	8.057	0.981	1984.52	2026.34	0.993
L1.4	2095	9.11	0.996	2330.032	2364.409	0.992
L1.5	1858	8.929	0.995	2043.658	2062.007	0.993
L1.6	1840	8.992	0.996	2056.238	2062.557	0.993
L1.7	1842	8.223	0.977	2016.137	2081.917	0.992
L1.8	1774	8.224	0.991	1956.331	1996.615	0.993
L3.1	1821	8.083	0.982	2024.978	2042.966	0.993
L3.2	1795	8.37	0.989	2068.583	2142.075	0.991
L3.3	1840	8.499	0.985	1946.26	1989.643	0.994
L3.4	1983	9.041	0.996	2125.714	2158.83	0.993
L3.5	1881	8.913	0.995	2099.04	2108.431	0.993
L3.6	1802	8.33	0.984	2017.967	2039.744	0.992
L3.7	1920	8.478	0.991	2113.05	2153.256	0.992
L3.8	1657	8.166	0.991	1815.935	1910.313	0.993
L7.1	1803	8.394	0.989	1975.117	2014.564	0.993
L7.2	1932	7.913	0.965	2139.964	2172.272	0.992
L7.3	1995	9.106	0.996	2181.75	2214.518	0.993
L7.4	1907	9.033	0.995	2052.102	2078.102	0.994
L7.5	1813	8.819	0.994	1968.058	2006.556	0.994
L7.6	1674	7.352	0.923	1862.445	1869.806	0.993
L7.7	1942	8.626	0.992	2124.527	2207.47	0.992
L7.8	1854	8.516	0.993	2089.18	2122.296	0.992
L14.1	1830	8.706	0.993	1978.892	1999.894	0.994
L14.2	1978	8.882	0.994	2144.472	2159.434	0.993
L14.3	1867	8.251	0.982	2062.27	2088.863	0.993
L14.4	1860	8.841	0.994	2016.552	2047.423	0.993
L14.5	2195	8.961	0.993	2407.686	2456.953	0.991
L14.6	1790	8.155	0.977	1991.254	2013.53	0.993
L14.7	1976	8.638	0.993	2218.519	2255.585	0.991

L14.8	1774	8.18	0.99	1993.247	2035.142	0.992

OTUs, Operational taxonomic units; ACE, Abundance-based coverage estimator.
